# P-948. Three Drugs, Two Pills, One Algorithm: HIV PrEP, PEP, and Treatment Initiation Consolidated Guidelines for Resource Limited and Non-Specialty Settings

**DOI:** 10.1093/ofid/ofae631.1138

**Published:** 2025-01-29

**Authors:** Colton P Boney, Erin G Park, Samantha V Hill, Robert W Parker, Andrew Jergel, Nicholas S Davis, Austin Yap

**Affiliations:** The Alabama College of Osteopathic Medicine, Huntsville, Alabama; Alabama College of Osteopathic Medicine, Gainesville, Florida; Emory University School of Medicine, Atlanta, Georgia; Alabama College of Osteopathic Medicine, Gainesville, Florida; Emory University School of Medicine, Atlanta, Georgia; Florida State University, Orlando, Florida; Howard University College of Medicine, Washington, District of Columbia

## Abstract

**Background:**

Accessing HIV prevention and treatment remains difficult throughout the United States (US). With the shortage of infectious disease physicians and other providers offering HIV prevention and treatment services, increasing the number of clinicians offering HIV Pre-Exposure Prophylaxis (PrEP), HIV Post-Exposure Prophylaxis (PEP), and Test & Treat (T&T) (where individuals testing positive for HIV are rapidly engaged in HIV treatment) is imperative. This project’s goal is to create a consolidated algorithm for HIV treatment and prevention that simplifies care for providers with few resources or limited experience.

The 3-2-1 Algorithm

**Figure:**
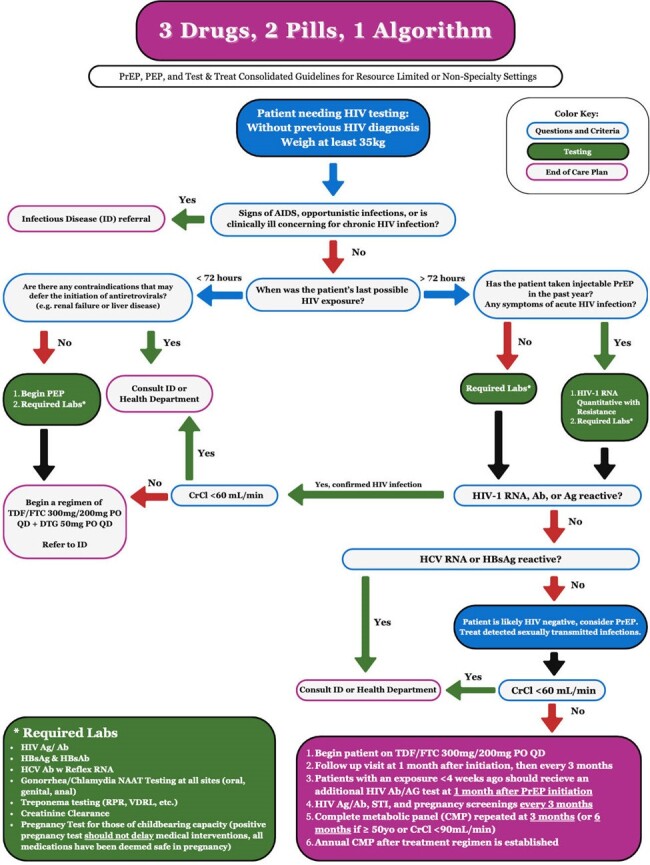

The algorithm includes the necessary steps, testing, and result interpretation to provide PrEP, PEP, and T&T services. Guidelines referenced in the making of this algorithm include those published the International Antiviral Society (IAS), the National Institutes of Health (NIH), and The New York State Department of Health AIDS Institute (NYSDOH AI).

**Methods:**

The algorithm was designed using published HIV care guidelines from multiple clinical bodies and includes PrEP, PEP, and T&T. Only three medications are offered in the algorithm to reduce cost of implementation. The combination of tenofovir disoproxil fumarate with emtricitabine provides low-cost, generic PrEP regardless of gender, pregnancy, or intravenous drug use while also providing the backbone needed for PEP and T&T. Dolutegravir, the third medicine, was chosen for PEP and T&T because of its once daily dosing and approval in pregnancy. The algorithm was tested with US medical students. Students completed an eight-question test unassisted then immediately repeated those questions with the algorithm. Questions included clinical scenarios related to HIV testing, PrEP, PEP, and T&T. Students were recruited by email for the online test. Pearson’s chi-squared compared pre vs post test scores; total score (number of items correct) used Paired Wilcoxon signed rank test.

**Results:**

76 students from 13 medical schools completed pre and posttest questions. Correctness increased from 3 of 8 (pretest) to 6 of 8 (posttest) (p=< 0.001). All questions increased in percentage correct with the algorithm. Change in correct responses on PEP, PrEP lab work, need for quantitative RNA with prior injectable PrEP use, and selecting a T&T regimen were all statistically significant. (p=< 0.001)

**Conclusion:**

This algorithm significantly improved medical students’ ability to accurately answer HIV clinical care questions and has the potential to expand access to HIV prevention and treatment. Further studies are needed to understand the algorithms’ effect in a clinical setting.

**Disclosures:**

**All Authors**: No reported disclosures

